# National automated surveillance of hospital onset bacteraemia and fungaemia using data from the national antimicrobial resistance surveillance system: a retrospective exploratory evaluation, the Netherlands, 2018 to 2023

**DOI:** 10.2807/1560-7917.ES.2026.31.28.2500881

**Published:** 2026-07-16

**Authors:** Manon Brekelmans, Tjallie van der Kooi, Annelot F Schoffelen, Rony E Zoetigheid, Maaike SM van Mourik, Sabine de Greeff, Stephanie van Rooden, on behalf of the ISIS-AR study group

**Affiliations:** 1 Centre for Infectious Disease Control, National Institute for Public Health and the Environment, Bilthoven, the Netherlands; 2 Department of Medical Microbiology and Infection Control, University Medical Centre Utrecht, Utrecht, the Netherlands; 3 The members of the ISIS-AR study group are listed in the Acknowledgements

**Keywords:** Automated surveillance, hospital-onset bacteraemia and fungaemia

## Abstract

**BACKGROUND:**

Hospital-onset bacteraemia and fungaemia (HOB) is a new target for automated surveillance of healthcare-associated infections.

**AIM:**

We aimed to evaluate (i) whether data from a national laboratory-based antimicrobial resistance (AMR) surveillance system (ISIS-AR) are a suitable source for fully automated HOB surveillance and (ii) whether the derived HOB rates from automated surveillance are a useful measure for national surveillance.

**METHODS:**

We applied an algorithm based on a consensus HOB definition to ISIS-AR data from 2018 to 2023. We performed: (i) technical evaluation of the data to determine their suitability for automated HOB surveillance, (ii) outcome validation of automatically identified HOB cases in four hospitals, and (iii) qualitative interviews with stakeholders on the anticipated value of HOB surveillance.

**RESULTS:**

Most parameters required for HOB identification were present, except for hospital admission dates (≥ 80% available in only 14 hospitals). Outcome validation showed accurate identification of HOBs caused by pathogens (non-commensals), but identification of HOBs caused by common commensals varied between hospitals, reflecting laboratory policy differences. Interviewees emphasised that feedback from national HOB surveillance to hospitals can be valuable for monitoring local trends, as signal for in-depth investigations and benchmarking.

**CONCLUSION:**

Dutch national automated HOB surveillance based on secondary use of national AMR surveillance data for pathogen-related HOBs is feasible, particularly if recording admission date is improved. Understanding local routine care and diagnostic practices is crucial for the interpretation of results. The main usefulness is expected at hospital level, while its value for national trend monitoring needs further study.

**Key public health message**

**What did you want to address in this study and why?**

This study focused on detecting bloodstream infections that arise in hospitals, known as hospital-onset bacteraemia and fungaemia (HOB) to find areas where infection prevention could be improved. We explored the possibility of reusing data from a national surveillance system for antimicrobial resistance to create a national HOB surveillance system with no or limited extra resources from hospitals.

**What have we learnt from this study?**

Our findings indicate that HOB cases can be identified using data collected for antimicrobial resistance surveillance. However, admission dates, an important data source for detecting HOBs, are missing in the majority of hospitals, which limits national coverage. Identification of HOB caused by skin flora varied due to differences in laboratory policies. Stakeholders were positive about this surveillance system as it would require only little extra effort for hospitals.

**What are the implications of your findings for public health?**

This study shows both the potential benefits and the challenges of using existing antimicrobial resistance surveillance data for HOB monitoring. This surveillance could serve as a starting point to gain more insight in the presence of HOB and to identify areas for improvement of infection prevention. Further, the added value of local trend monitoring and benchmarking is expected at hospital level, while the potential for public health needs further study.

## Introduction

Surveillance of healthcare-associated infections (HAIs) is a key component of effective infection prevention programmes in hospitals [1]. Automated surveillance is defined as any form of surveillance where manual assessment of infection cases is (partly) replaced by an automated process, using routine care data [2]. This can reduce workload and provide more consistent, standardised results compared with manual methods [2-6].

Hospital-onset bacteraemia and fungaemia (HOB) captures all bloodstream infections (BSI) including secondary BSI and BSI related to catheters, in patients admitted to the hospital, providing an objective and extensive measure of HAI that is suitable for automated surveillance [7-11]. However, the relevance of HOB surveillance as a tool to help prevent HAI requires further study [7,12]. The European PRAISE (Providing a Roadmap for Automated Infection Surveillance in Europe) network [2] published a detailed, standardised HOB definition and the description of an algorithm for fully automated case detection, accompanied by a minimum dataset (PRAISE-MDS) of source data from electronic health records [12]. In the Netherlands, many of the elements of the source data required for fully automated HOB surveillance are already systematically collected at the National Institute for Public Health and the Environment (RIVM) in the Dutch Infectious Disease Surveillance Information System for Antimicrobial Resistance (ISIS-AR). The ISIS-AR gathers positive microbiological culture results that include routine antimicrobial susceptibility testing (AST) data in order to monitor antimicrobial resistance (AMR) and outbreaks involving AMR [13]. If these ISIS-AR data are suitable as a source for HOB surveillance, a nationwide HOB surveillance could be implemented relatively easily within the national HAI surveillance of the RIVM (the PREZIES network), with no or limited extra resources from hospitals.

We aimed to evaluate (i) whether data from the ISIS-AR currently in operation are a suitable source for fully automated HOB surveillance and (ii) whether the derived HOB rates from automated surveillance are considered a useful measure for (national) surveillance.

## Methods

### Study design and setting

In this exploratory retrospective study, we systematically evaluated the concepts from the United States (US) Centers for Disease Control and Prevention (CDC) Updated Guidelines for Evaluating Public Health Surveillance Systems [14] to assess automated HOB surveillance with readily available data as a source, using both quantitative and qualitative methods, as outlined in Table 1. The evaluation consisted of three elements: (i) a technical evaluation of the data to determine fit for purpose, (ii) outcome validation of identified HOB cases in four hospitals, and (iii) a qualitative evaluation to assess the value of HOB surveillance in general and based on ISIS-AR data. This study was conducted in the Netherlands with data from 2018 to 2023. As the hospital and laboratory landscape changes over time through mergers between hospitals and/or laboratories, the exact number of hospitals and laboratories in the Netherlands during this study period cannot be given. At the end of the study period, the Netherlands had ca 75 hospitals, of which eight were university medical centres. Individual hospitals could have multiple locations.

**Table 1:** Design of the evaluation framework for hospital-onset bacteraemia and fungaemia surveillance based on the United States Centers for Disease Control Updated Guidelines for Evaluating Public Health Surveillance Systems [14], the Netherlands, 2018–2023

Task	CDC guideline [14]		Application within study		
	Description	Definition	Level of evaluation	Study part	Methods
A	Engage the stakeholders in the evaluation	Those persons or organisations who use data for the prevention and control of infections	NA	Qualitative evaluation	Inclusion of stakeholders in our interviews
B	Describe the surveillance system to be evaluated				
B.1	Public health importance of health-related event under surveillance	Indices of frequency, severity; costs; preventability etc	Surveillance system	Descriptive based on literature and qualitative evaluation	Introduction and HOB definition; additional evaluation of importance in interviews
B.2	Describe the purpose and operation of the surveillance system	Purpose, uses of data, case definition, flowchart, components etc	Surveillance system	Descriptive	Description of the surveillance system with a flowchart
B.3	Describe the resources used to operate the surveillance system	Describe funding sources, personnel requirements, and other resources	Surveillance system	Descriptive	The proposed surveillance system can re-use data from an existing surveillance programme and therefore the required additional resources will be limited
C	Focus the evaluation design	The direction and process of the evaluation must be focused to ensure that time and resources are used as efficiently as possible	NA	Aim of study	This study focusses on the data technical evaluation and the evaluation of the perceived usefulness of the proposed surveillance system with relevant stakeholders
D	Gather credible evidence regarding the performance of the surveillance system				
D.1	Indicate the level of usefulness	If it contributes to the prevention and control of HAI, including an improved understanding of the public health implications of HAI; If it helps to determine that an HAI previously thought to be unimportant is actually important; useful if contributing to performance measures	Source data and surveillance system	Qualitative evaluation	Interviews
D.2	Describe each system attribute				
D.2.a	Simplicity	Structure and ease of operation of the surveillance system	Surveillance system	Data technical evaluation	The data flow and process are visualised using a flowchart and described
D.2.b	Flexibility	Adapt to changing information needs or operating conditions with little additional time, personnel, or allocated funds	Surveillance system	Data technical evaluation	Description of steps that need to be taken in case of changes in the case definition or gathering additional information
D.2.c	Data quality	Completeness and validity of the data	Source data	Data technical evaluation	Availability and completeness of the ISIS-AR data and denominator data, with the PRAISE MDS as reference framework
D.2.d	Acceptability	The willingness of persons and organisations to participate in the surveillance system	Surveillance system	Qualitative evaluation	Interviews
D.2.e	Sensitivity	Proportion of cases detected by the surveillance system and ability to detect outbreaks, including the ability to monitor changes in the number of cases and infection rate over time	Surveillance system	Outcome validation and Qualitative evaluation	Comparison of surveillance results with reference standard (case reports) including correlation analysis and assessment of trends by stakeholders from interviews
D.2.f	Positive predictive value	Proportion of reported cases that actually have a HOB	Surveillance system	NA	Not possible since data could not be linked on a patient level
D.2.g	Representativeness	Accurately describes the occurrence of HOB over time and its distribution in the population by place and person	Source data	Data technical evaluation	The percentage of hospitals in ISIS-AR providing sufficient data to comply with the MDS of the PRAISE consensus definition (for at least 80% of the isolates). The available baseline characteristics (age, sex, hospital type, ward type) are compared between the total ISIS-AR data and the subgroup that provided sufficient data
D.2.h	Timeliness	Speed between steps in the surveillance system	Source data and surveillance system	Data technical evaluation	The frequency of data submissions to ISIS-AR and the publication timing of denominator data will be described, and the time needed to perform the surveillance
D.2.i	Stability	Reliability and availability of the surveillance system	Source data	Data technical evaluation	The yearly percentage of laboratories and hospitals participating in ISIS-AR, and eligible for HOB surveillance, continuity of participation, and yearly availability of denominator data will be described
E	Justify and state conclusions, make recommendations	NA	NA	NA	Conclusion and discussion of scientific publication
F	Ensure use of evaluation findings and share lessons learnt	NA	NA	NA	Scientific publication; results of this study will be included in the decision process whether to actually implement HOB surveillance based on ISIS-AR data

CDC: Centers for Disease Control and Prevention; HAI: healthcare-associated infections; HOB: hospital-onset bacteraemia and fungaemia; ISIS-AR: Dutch Infectious Disease Surveillance Information System for Antimicrobial Resistance; NA: not applicable; PRAISE MDS: minimum dataset from the PRAISE (Providing a Roadmap for Automated Infection Surveillance in Europe) network.

### Hospital-onset bacteraemia and fungaemia definition

We aligned our definition with the consensus definition of HOB as developed by the PRAISE network [12], hereafter referred to as the PRAISE consensus definition. In short, HOB is defined as a positive blood culture, taken 2 or more days after admission, with a maximum episode duration of 14 days, or until the end of the hospital stay. Microorganisms are classified as either pathogens (non-commensals) or common commensals. If a common commensal (e.g. coagulase-negative staphylococci or species of bacilli and streptococci), as defined by the CDC [15] is identified, a second positive blood culture containing the same microorganism within 2 days is required. If multiple microorganism episodes start within 2 days of each other, these are taken together and the HOB is considered polymicrobial.

#### Description of data sources for hospital-onset bacteraemia and fungaemia surveillance output

**Figure 1:**
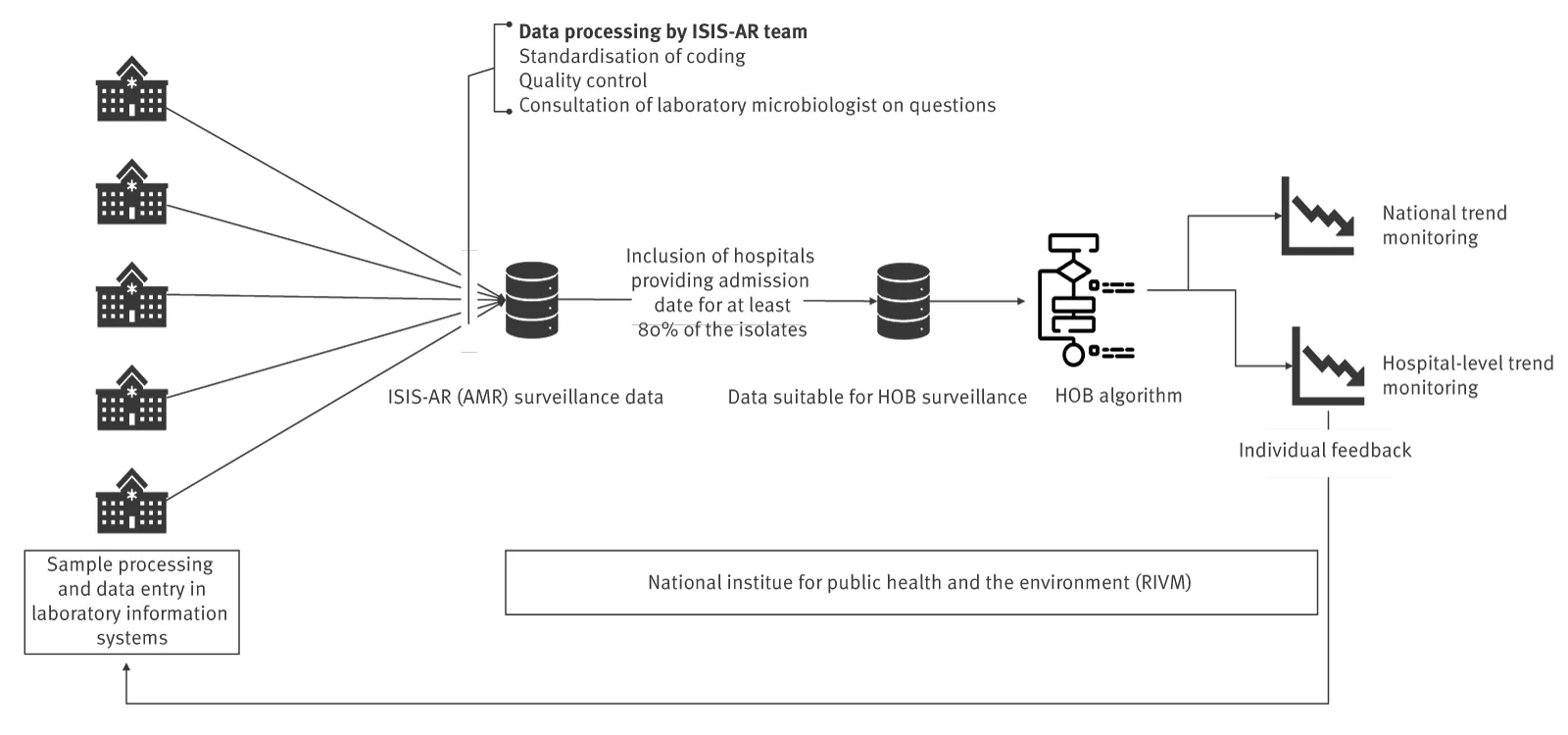
Data flow of the proposed national hospital-onset bacteraemia and fungaemia surveillance system based on routine antimicrobial resistance surveillance, the Netherlands, 2018–2023

AMR: antimicrobial resistance; HOB: hospital-onset bacteraemia and fungaemia; ISIS-AR: Dutch Infectious Disease Surveillance Information System for Antimicrobial Resistance.

For this national HOB surveillance system under evaluation, the ISIS-AR data served as a data source. In the ISIS-AR, detailed routinely available microbiological data of positive cultures from clinical laboratories are collected if AST has been performed, including information on bacterial species, sample material, AST results and patient demographics (year and month of birth, sex and four-digit postal code). The ISIS-AR surveillance system features a well-established data infrastructure including translation to standardised codes (SNOMED CT [16] and LOINC codes [17]) before data submission and quality control [13] (Figure 1).

A laboratory microbiologist is consulted to answer any questions that may come up following the quality control.

Denominator data (total number of hospital admissions) were collected on an annual basis from an open-source national dataset containing the denominator data at hospital level, which was available only until 2021. More detailed denominator data were not available from an open-source data source. Denominator data for 2023 were supplemented with the number of hospital admissions from ‘Ziekenhuischeck’ (https://www.ziekenhuischeck.nl) and intensive care unit (ICU) admissions from the National Intensive Care Evaluation (NICE) (www.stichting-nice.nl), both open-source hospital datasets. The denominator for 2022 was not openly available and therefore estimated by averaging denominator data from 2021 and 2023.

### Data collection and analysis

#### Technical evaluation of the data

Hospitals were considered eligible for inclusion in the automated HOB surveillance system when they provided hospital admission dates for at least 80% of the isolates in the reporting period, as this threshold ensured a balance between including enough hospitals and maintaining adequate completeness of admission dates. Relevant data for this HOB surveillance were collected from the ISIS-AR for the period 2018–2023 and included culture and AST results from all positive diagnostic blood cultures collected during hospital admission.

According to the CDC guidelines (Table 1, section D), completeness, representativeness, timeliness and stability of the source data, i.e. ISIS-AR data, were described, as well as the simplicity and flexibility of the HOB surveillance system. Statistical tests for differences in proportions and medians were performed using chi-square and Mann–Whitney tests, respectively. A p value of less than 0.05 was considered statistically significant. For eligible hospitals, HOB episodes (numerator data) were calculated per year, per hospital ward type (ICU, non-ICU) and per microorganism and the incidence over time was calculated as the number of HOBs per 100 admissions. These HOB rates were evaluated for trends (Figure 1). Analyses were performed using SAS version 9.4 (SAS Institute, Cary, US).

### Outcome validation

As part of the data quality evaluation, HOB surveillance outcomes were validated. Aggregated HOB counts of four hospitals were compared with aggregated HOB counts determined in a previous study where automated HOB surveillance was based on data directly extracted from electronic health records (EHR) in these four hospitals (one university hospital (including a children’s hospital) and three top clinical hospitals) in the region of Utrecht (reference standard) [18]. Aggregated HOB numbers (absolute counts) were compared visually for each hospital and year, for pathogens and common commensals separately for the period 2018–2021. In addition, we calculated the differences in HOB counts between these groups per hospital compared with the reference data. Since the data could not be linked at patient level, sensitivity and positive predictive value, as defined in the CDC guidelines, (Table 1 Sections D2.e and D2.f) could not be calculated.

### Qualitative evaluation

We performed semi-structured interviews with stakeholders who were recruited through an invitation in the PREZIES newsletter and by snowball sampling where interviewees were contacted in person or by email. We aimed for a heterogeneous group of stakeholders, representing both local and national perspectives, including infection control practitioners, medical microbiologists, infectious diseases physicians, internal medicine physicians and epidemiologists from across the country (for detailed information on interviewees, see Supplement S1). An interview guide (details shown in Supplement S2) with an emergent design was developed to assess (i) the acceptance and usefulness of HOB in principle as an HAI target, (ii) the usefulness of HOB in a national surveillance system, and (iii) suitability of the ISIS-AR as a data source for national HOB surveillance and the validity of the calculated national HOB trends. The interviews were conducted by the researchers (MB and TvdK) either face-to-face or by video call. We initially planned 10 interviews of ca 1 hour and continued until all stakeholder groups were included and data saturation was reached. Interviews were recorded and transcribed verbatim by a contracted transcription agency. Qualitative data were coded with a flexible approach that combined deductive and inductive strategies for thematic analysis [19]. Coding was performed by two researchers independently (MB and TvdK) using MAXQDA 26 (VERBI software, Berlin, https://www.maxqda.com). When no consensus was reached, a third researcher was consulted (SR). The Consolidated criteria for Reporting Qualitative research (COREQ) checklist [20] was the foundation for reporting the qualitative methods, analyses and results.

## Results

### Technical evaluation of the data

#### Simplicity and flexibility

A HOB surveillance system based on ISIS-AR data as a source benefits from an existing infrastructure (Figure 1) and well-established data governance, making this system relatively simple to implement. However, as the ISIS-AR relies exclusively on data extracted from the laboratory information system for the purpose of AMR surveillance, the system is not very flexible as additional data are not instantly available for other purposes outside the primary scope of the ISIS-AR. However, the algorithm for HOB detection was applied centrally, and was therefore the same for all hospitals. In addition, adaptations to the algorithm can be easily implemented, ensuring flexibility of case definitions.

#### Data quality

The quality of the data in the ISIS-AR is ensured by monthly data quality and integrity checks, including format validation and completeness and plausibility checks, e.g. the proportions of cultured organisms, or reported reason for culturing (i.e. screening, diagnostic) and unusual test values.

In Table 2 and Supplement S3, the availability and completeness of the PRAISE MDS variables in the ISIS-AR data are presented. Most of the mandatory variables are 100% available. However, admission date, a non-mandatory item in the ISIS-AR but a prerequisite for the identification of HOB, was provided for only 64% of all isolates and 79% of ICU isolates (Table 2). Additionally, denominator data, i.e. the number of admissions per year from the initial source, were only available until 2021. Another source was limited to the previous calendar year, resulting in missing data for 2022. Data on negative cultures needed to determine blood culture frequency are not collected in the ISIS-AR. Information on patient transfers within the hospital is not available. This implies that the attributable ward, as defined by the PRAISE consensus definition, cannot be determined, but data on the sample ward are available.

**Table 2:** Quality of the Dutch Infectious Disease Surveillance Information System for Antimicrobial Resistance data for hospital-onset bacteraemia and fungaemia surveillance based on the minimum dataset from the PRAISE (Providing a Roadmap for Automated Infection Surveillance in Europe network), the Netherlands, 2018–2023

PRAISE MDS	ISIS-AR data	
Variable	Availability	Completeness
Patients		
Patient ID	Yes	100%
Sex	Yes	100%
Birth Date	Partial	100% for birth year and month
Blood cultures		
Blood sample ID	Yes	100%
Sample date	Yes	100%
Sample ward	Yes	100% for sample ward or order ward
Isolate number	Yes	100%
Microorganism (local names)	Yes	100%
Attributable ward (i.e. ward where patient was 2 days before culture sample taken)	No	NA
Hospital admission date	Partial	273,883/428,360 (64%) of all isolates have an admission date 51,145/64,739 (79%) of all ICU isolates have an admission date

ICU: intensive care unit; ID: identification; ISIS-AR: Dutch Infectious Disease Surveillance Information System for Antimicrobial Resistance; PRAISE MDS: minimum dataset from the PRAISE (Providing a Roadmap for Automated Infection Surveillance in Europe network); NA: not applicable.

### Stability and representativeness

Data from the ISIS-AR during the period 2018–2023 included 428,360 blood culture isolates from 50 different laboratories serving the large majority (ca 80%) of hospitals in the Netherlands. On an annual basis, 26 to 33 hospitals provided sufficiently complete admission dates and could be included in HOB surveillance. When focusing only on hospital admission dates for ICU-related blood cultures, the data appeared more complete; ICU departments of 38 to 48 hospitals could be included in the study period. In these samples, academic hospitals were overrepresented compared with the complete ISIS-AR data (41% vs 25%, respectively) and general hospitals were underrepresented compared with the complete ISIS-AR data (17% vs 26%, respectively). Otherwise, although the differences were significantly different due to the large numbers, the samples were comparable in terms of age, sex, ICU and medical specialty distribution (Table 3, for absolute numbers see Supplement S4).

**Table 3:** Stability and representativeness of the Dutch Infectious Disease Surveillance Information System for Antimicrobial Resistance data and data suitable for hospital-onset bacteraemia and fungaemia surveillance, the Netherlands, 2018–2023

CDC guideline concept	Characteristic	ISIS-AR data 2018–2023		Data eligible for HOB surveillance 2018–2023 (> 80% of admission dates available)	
		Total ISIS-AR data	Selection of hospitals consistently included in ISIS-AR data^b^	Annual surveillance-suitable data	Selection of hospitals with consistent surveillance-eligible data^b^
	Number of records (n)	428,360	388,114	171,454	136,175
**Stability**	**Number of hospitals by year**				
	2018	64	54 (8 academic, 26 top clinical and 20 general hospitals)	32	14 (6 academic, 4 top clinical and 4 general hospitals)
	2019	60		26	
	2020	66		33	
	2021	65		32	
	2022	66		30	
	2023	71		28	
**Representativeness (blood culture isolate data)^a^**	**Birth year** (median (Q1–Q3))	1950 (1941–1963)	1950 (1941–1963)	1954 (1944–1969)	1955 (1945–1973)
	**Sex** (%)				
	Males	60	60	61	61
	Females	40	40	39	39
	**Hospital type** (%)				
	General	26	25	17	13
	Top clinical	49	50	43	35
	Academic	25	24	40	52
	**Ward type** (%)				
	ICU	15	15	18	20
	Non-ICU	85	85	82	80
	**Medical specialty** (%)				
	Internal medicine	28	27	23	20
	ICU	9	9	10	12
	Surgery	9	9	8	9
	Pulmonary diseases	7	7	6	5
	Emergency department	7	7	8	4
	Cardiology	5	5	5	4
	Urology	4	4	3	3
	Gastro-enterology	4	4	4	4
	Oncology	3	3	5	6
	Paediatrics	3	3	3	3
	Neurology	3	2	2	2
	Haematology	2	2	3	6
	Other	16	18	18	22

CDC: Centers for Disease Control and Prevention; HOB: hospital-onset bacteraemia and fungaemia; ICU: intensive care unit; ISIS-AR: Dutch Infectious Disease Surveillance Information System for Antimicrobial Resistance; Q: quartile.

^a^ Percentages based on all positive blood culture isolates from the study period.

^b^ Consistent data: data continuously available in all 6 years of study period.

All differences between the total ISIS-AR dataset and the annual surveillance suitable data, and between the selection of hospitals consistently included in ISIS-AR data and selection of hospitals with consistent surveillance-eligible data are statistically significant (p < 0.0001).

A total of 54 hospitals consistently provided data during the 6-year study period to the ISIS-AR (eight academic, 26 top clinical and 20 general hospitals). However, only 14 hospitals (six academic, four top clinical and four general hospitals) of these 54 hospitals provided sufficient admission dates throughout the study period and were therefore eligible for HOB surveillance. Academic hospitals were overrepresented in the consistent surveillance-eligible data compared with the consistent ISIS-AR dataset (52% vs 24%, respectively). Additionally, for the distribution of medical specialties for which patients were admitted, there were some differences between the consistent surveillance-eligible data and the consistent ISIS-AR dataset: relatively fewer isolates were related to internal medicine (20% vs 27%) and the emergency department (4% vs 7%), but more isolates were related to the ICU (12% vs 9%), oncology (6% vs 3%) and haematology (6% vs 2%) (Table 3).

### Timeliness

Timeliness is mainly dependent on data processing for the ISIS-AR, where data are collected through near real-time Health Level Seven (HL7) messages or monthly manual data submission. It is estimated that with this manual data collection and quality procedures, HOB reporting would have a maximum delay of 2 months.

#### Outcome validation

**Figure 2:**
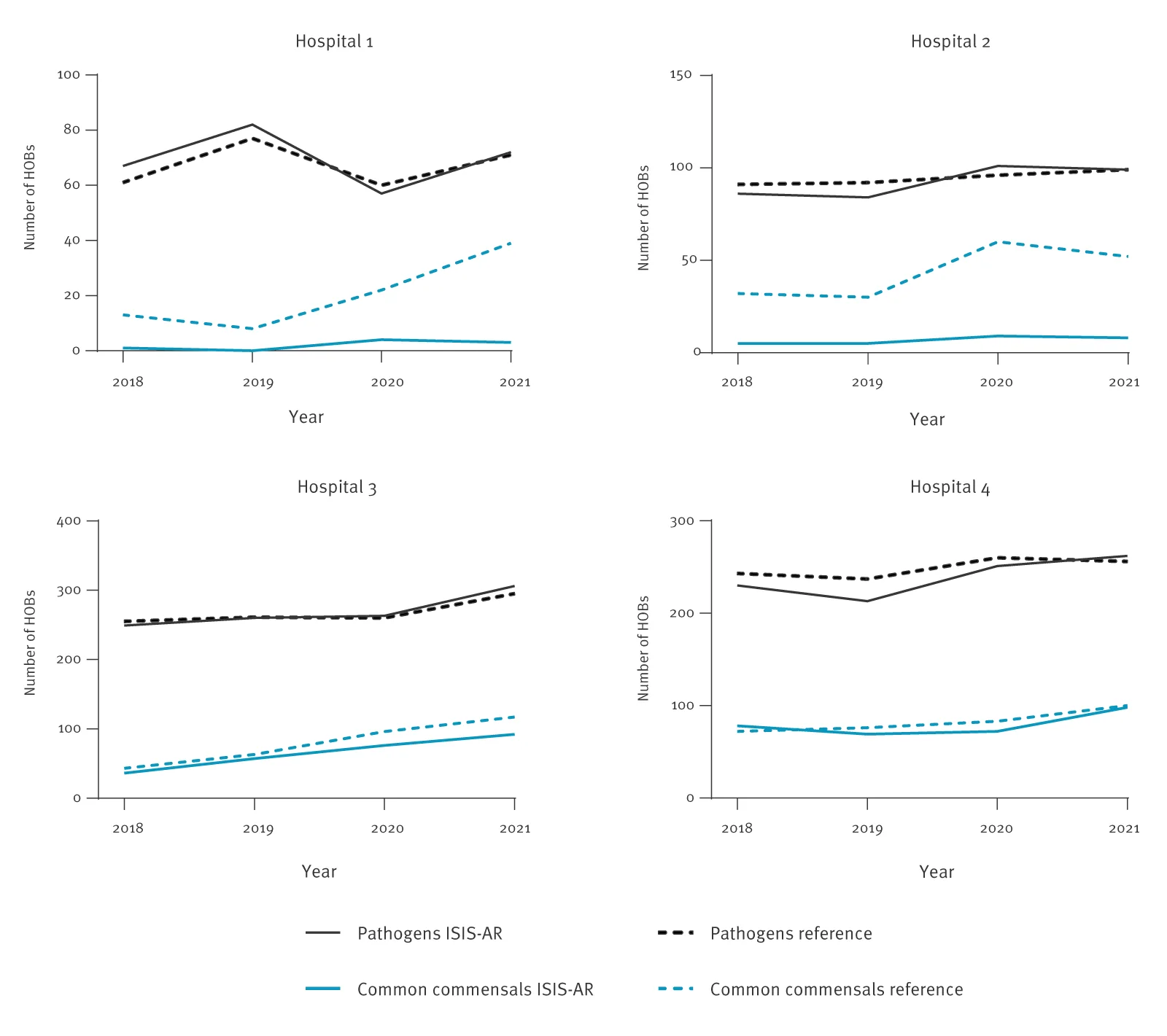
Trends in absolute number of hospital-onset bacteraemia and fungaemia cases detected with automated surveillance based on the Dutch Infectious Disease Surveillance Information System for Antimicrobial Resistance vs electronic health records for common commensals^a^ and pathogens (non-commensals) in four hospitals, the Netherlands, 2018–2023

HOB: hospital-onset bacteraemia and fungaemia; ISIS-AR: Dutch Infectious Disease Surveillance Information System for Antimicrobial Resistance.

^a^ Common commensals are defined based on the Centers for Disease Control and Prevention classification [15]. Admission dates were reported for 42% of the positive isolates in hospital 1, 75% in hospital 2, 99.5% in hospital 3 and 76% in hospital 4.

Among the four hospitals included in the outcome validation, there was some variety in the completeness of the admission dates: 42%, 75%, 99.5% and 76%, respectively, for Hospitals 1,2,3 and 4. Despite missing admission dates, the number and trend of HOBs caused by pathogens based on ISIS-AR data were comparable to the number using HOB-EHR (reference data). In Hospital 1, 353 HOBs were found using the ISIS-AR data and 350 using HOB-EHR (0.9% difference). For Hospital 2, 370 vs 378 HOB cases were identified (-2.1% difference). For Hospital 3, 1,078 vs 1,071 cases were identified (0.7% difference) and for Hospital 4, 1,245 vs 1,266 cases (-1.7% difference). However, discrepancies were observed in the number of HOBs caused by common commensals in Hospital 1 and 2 (Figure 2). In Hospital 1, 10 HOBs were identified using the ISIS-AR data and 95 using HOB-EHR (–89.5% difference), while for Hospital 2, 27 vs 174 cases, respectively, were identified (–84.5% difference). For Hospitals 3 and 4, the absolute numbers were comparable: for Hospital 3, 261 ISIS-AR data HOBS vs 319 HOB-EHR (–18.2% difference) and for Hospital 4, 446 ISIS-AR data HOBs vs 462 HOB-EHR (–3.5% difference). Comparisons by microorganism group are presented in Supplement S5. Additional enquiries with the four laboratories clarified differences in policies regarding routine AST procedures; Hospitals 1 and 2 conducted AST for common commensals only in selected cases, whereas Hospitals 3 and 4 did so routinely for all positive blood cultures. As only AST-tested isolates are reported to the ISIS-AR, this affected the ability to detect HOBs caused by common commensals, since the requirement of two positive cultures was not always fulfilled.

### Qualitative evaluation

#### Interview participants

We performed 12 interviews: four with infection control practitioners, three with medical microbiologists, three with internal medicine physicians and two with epidemiologists (for detailed information on interviewees, see Supplement S1).

#### Acceptance and usefulness of hospital-onset bacteraemia and fungaemia surveillance in principle as healthcare-associated infection outcome

Overall, HOB was generally regarded as a relevant HAI outcome and automated HOB surveillance was considered an efficient signalling system for detecting increases in HOB rates. Subsequent in-depth investigations into the aetiology of HOB could lead to quality improvement within hospitals (see Quote 1). In general, HOBs with catheter-related bacteraemia as a source were mostly considered preventable; the disease burden and preventability of other causes would require further research. Although the source of an HOB in surveillance is considered highly relevant, including this in surveillance would need to be balanced against the additional workload.

Quote 1: “*We look, of course, is it a real increase, and in case of a positive case, is there something going on?*” Infection control practitioner, large hospital.

Overall, the PRAISE consensus definition was accepted, although there was some discussion regarding episode duration and the hospital-onset period, which may not always apply to specific clinical indications or individual cases, as well as the confirmation of common commensals, which depends on local blood culture practices. Most respondents agreed that some arbitrary choices are inevitable in order to achieve standardised surveillance outcomes.

### National hospital-onset bacteraemia and fungaemia surveillance for national trend monitoring

National surveillance could reveal developments that remain unnoticed at hospital level, although the value needs to be demonstrated (e.g. evaluation of policy changes) (see Quote 2). For correct interpretation, insight into changes in, for example, complexity of care or clinical processes, is needed.

Quote 2: *“… and I think policy makers require these kind of (national) numbers to evaluate if should we address this (HOB) more or is it something we are currently doing well.”* Medical microbiologist, large hospital.

However, all respondents were of the opinion that national trend monitoring was not the main added value of a national surveillance system for HOB, but rather local trend monitoring and benchmarking (see Quote 3). Benchmarking is useful when reliable comparisons can be made, with stratification by hospital type, medical speciality or ward type and microorganism.

Quote 3: “*If I look purely into our local practice and what I am doing it for, then it is for here (this hospital), so to say, and to be able to guide our infection prevention policy. And not nationally, to make choices in policy or stewardship. Of course, they are related to each other. So initially, I think it is primarily for one’s own hospital, but also benchmarking, especially with other academic hospitals that have a similar patient population, does provide information.”* Infection control practitioner, large hospital.

### National hospital-onset bacteraemia and fungaemia surveillance for local trend monitoring

**Figure 3:**
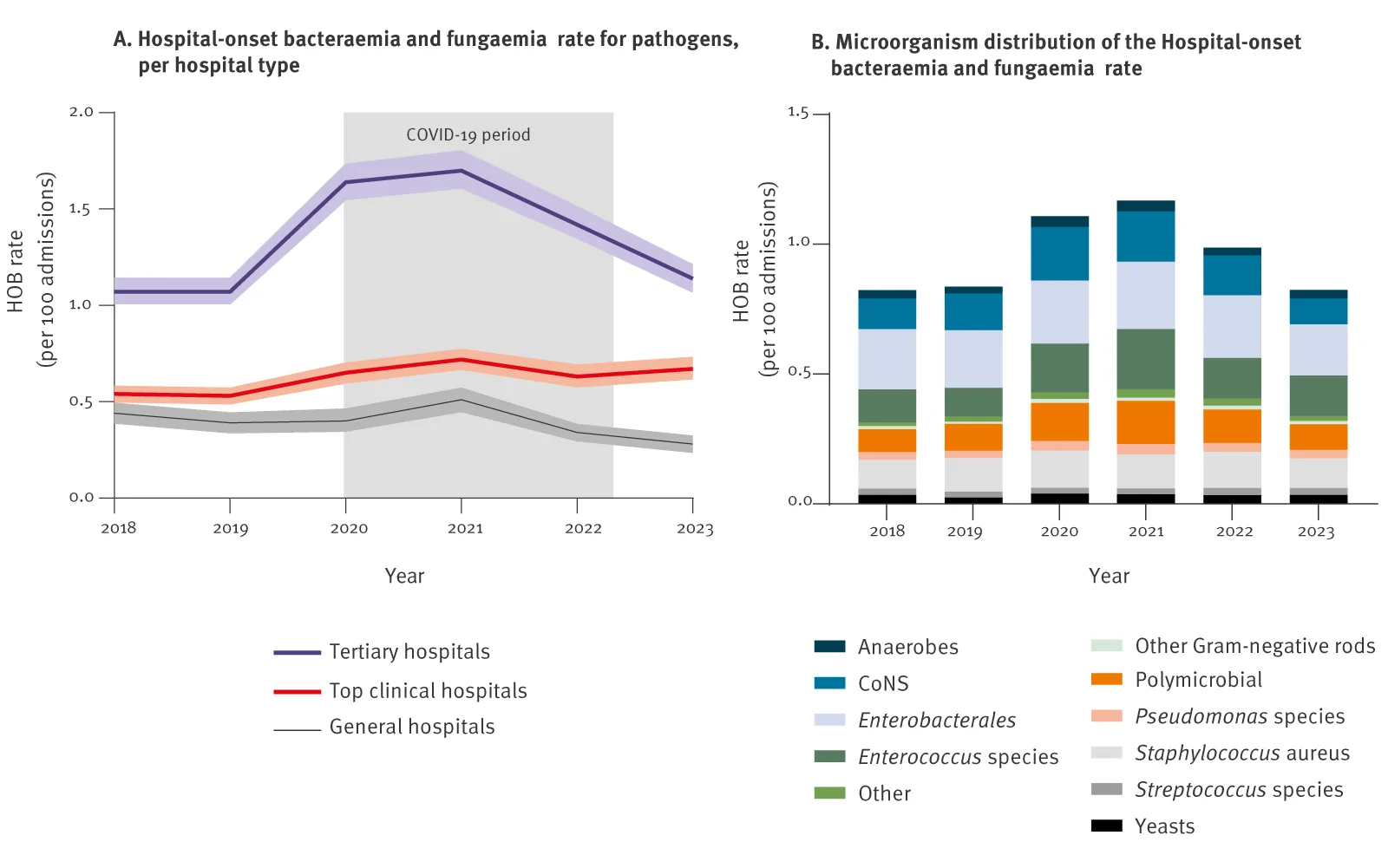
National trends of hospital-onset bacteraemia and fungaemia over time, calculated from automated surveillance using national antimicrobial resistance surveillance data, the Netherlands, 2018–2023 (n = 14 hospitals)

CoNS: coagulase-negative staphylococci; HOB: hospital-onset bacteraemia and fungaemia.

The trends, as shown in Figure 3, were recognised by all participants and also observed in their local setting, particularly the increase in HOB rates with pathogens during the COVID-19 pandemic period (2020–2022), and the differences between hospital types.

Panel A: bands represent 95% confidence intervals. Note: these confidence intervals were not shown during interviews.

Panel B: of note, common commensals are included in this graph despite underestimation of HOBs caused by common commensals. *Enterococcus* species are classified as pathogens.

Feedback of local results from a national HOB surveillance system (i.e. number of HOBs, HOB rates by stratification) is valuable but should be detailed and timely. Ideally, the feedback is as detailed as possible, but the number of HOBs should be sufficiently large to interpret trends, and the benefits should outweigh the effort (see Quote 4). The infection prevention and control (IPC) department was considered the department primarily working with HOB surveillance results, whereas opinions on the involvement of clinical departments, quality departments and management differed across hospitals.

Quote 4: “*If you want it for infection prevention, that can use these data to look at: hey, where do we see a trend break, and is it something that should be investigated at a local level? – then I think: the more detailed feedback, the better.*” Internal medicine physician, large hospital.

### Use of the Dutch Infectious Disease Surveillance Information System for Antimicrobial Resistance data for a national hospital-onset bacteraemia and fungaemia surveillance system

Participants highlighted the advantages of reusing existing source data, such as ISIS-AR data, particularly the minimal workload for hospitals and the application of a uniform algorithm across hospitals (see Quote 5).

Quote 5: “*At least, it (data from ISIS-AR) makes it possible to collect data in a standardised way from many hospitals. I see that as the major advantage. And also that you don’t create extra work for the hospitals to achieve that.”* Epidemiologist at national level.

For local use, stratification by ward or medical specialty, even in absolute numbers, would be valuable. Reported limitations of ISIS-AR data as a source included the restricted level of detail of patient and ward characteristics for reporting and the limitation to HOBs caused only by pathogens. Interviewees indicated that it was considered feasible to provide admission dates and the total number of patient admissions, thereby improving coverage, especially when supported by examples from other hospitals. As prerequisites for useful HOB surveillance, participants mentioned the importance of understanding the data and data quality for proper interpretation. Reidentification of patients with a HOB in local systems was considered necessary for further evaluation.

## Discussion

In this study, we systematically evaluated the feasibility of a national surveillance system for HOB based on data from an existing AMR surveillance system in the Netherlands - the ISIS-AR. Most variables required for HOB identification, as stated by the PRAISE consensus definition, were available and complete in the ISIS-AR data, except for admission date, which was available only for a limited number of hospitals. Outcome validation from four hospitals showed that the number of HOBs caused by pathogens were comparable to the reference method, whereas HOBs caused by common commensals were under-reported. The qualitative evaluation highlighted the potential usefulness of automated HOB surveillance - it could serve as a signal for hospitals to initiate in-depth local investigations to better identify specific areas where interventions to improve infection prevention and control are needed. Benchmarking and local trend monitoring through timely feedback were seen as the main value of a national HOB surveillance. Interpreting changes in HOB rates requires a clear view of care practices and patient populations. As HOB is a hospital-wide target, distinguishing true changes in incidence from shifts in these factors is even more critical than for existing HAI targets and will require attention when reporting formats are designed. The minimal workload for hospitals achieved by reusing ISIS-AR data are seen as a clear advantage.

However, attention to some limitations, considerations and remaining questions regarding the studied automated HOB surveillance is warranted. Firstly, our study showed under-reporting of HOBs caused by common commensals; in some hospitals AST was not performed routinely when contamination was suspected, resulting in an under-reporting of HOB caused by common commensals based on ISIS-AR data. As only four hospitals were included in the outcome validation, there is a possibility that other laboratory policy differences affected the results in unknown ways. Although the interpretation of existing data may appear straightforward, a thorough understanding of the source data, hospital and laboratory procedures, and their interpretation is essential to ensure outcome comparability and validity in large-scale surveillance. Alternatively, focus [11] or other case-definitions not including the confirmation of common commensals [8,21,22] could be considered, but this would affect international comparability.

Secondly, as a fully laboratory-based system, it lacks some clinical patient information, such as risk factors, hospital- or ward-specific admission dates and comprehensive denominator data. Despite this limitation, reusing ISIS-AR data with high, but not full, national coverage offers a strong foundation for automated HOB surveillance. Importantly, however, the limited availability of hospital admission dates, a non-mandatory variable in the ISIS-AR, with academic hospitals consequently overrepresented, affects national representativeness. However, one of the validation hospitals had only 42% completeness of recorded admission dates, yet reported a similar number of HOBs caused by pathogens as was provided by EHR reference data. This warrants further investigation, as patients with or without a recorded admission date may represent a specific subgroup. In this specific hospital, differences in completeness of recorded admission dates were observed between isolates from different medical specialties. In other hospitals, other causes and effects of missing admission dates could be present. Therefore, future initiatives should systematically assess the completeness of recorded admission dates and stimulate improvement where needed. In addition, completeness of admission date data should be routinely monitored when interpreting surveillance results, effectively incorporating a data quality check into the analysis. Monitoring changes in completeness over time is essential, as variation in data completeness may complicate comparisons with historical data and affect trend analysis. Interviewees indicated that sharing admission dates and total numbers of patient admissions should be feasible, and the prospect of receiving hospital-specific feedback on HOB trends may serve as an incentive. In addition, extra variables such as source of HOB or device use could improve interpretability and support more targeted interventions, but the feasibility of collecting these data, possibly using different data sources or methods, requires further study.

Thirdly, benchmarking was mentioned as the main added value of a national system. A typical benchmark is comparison at medical specialty or ward level between hospitals of the same hospital type, as indicated by interviewees. Using ISIS-AR as a data source, it is possible to identify HOBs at medical specialty or ward or level, but due to the lack of appropriate denominator data at ward level, incidence rates can only be calculated by distinguishing between ICU and non-ICU wards within a hospital. Further, especially for hospital-wide targets such as HOB, as opposed to more specific HAI targets (e.g. surgical site infections (SSI) or central line-associated bloodstream infections (CLABSI)) [11,23], additional case-mix adjustment should also be considered. This would require in-depth research to select appropriate risk factors for risk adjustment, as this determination is far from straightforward [24-26]. It would also primarily involve the collection of additional characteristics and source data, and the possibilities using ISIS-AR data are relatively limited. Other surveillance infrastructures that allow for analyses of more routine care data could offer a solution if more detailed data were needed.

Our findings show that data from a nationally representative laboratory-based AMR surveillance system (ISIS-AR) provide a pragmatic and scalable starting point for national automated surveillance of HOB, even without full EHR integration. Despite limitations, particularly incomplete recording of admission dates, this approach enables early implementation. This, in turn, allows important insights to be gained into the source data and its implications for quality assurance, comparability of outcomes and the interpretation and actionability of HOB surveillance outcomes. As such, our approach can serve as an intermediate step towards a surveillance system applied directly to EHR data.

When implemented, lessons could be learnt on the extent to which HOB surveillance delivers actionable data that support prevention efforts and contribute to national policy decisions. Moreover, at hospital level, it could be evaluated how and when the outcomes of this HOB surveillance system guide local IPC actions, what interventions could be implemented and what effects these measures have on HOB rates. Setting up an automated HOB surveillance system was anticipated to be useful, particularly for monitoring local trends and benchmarking in the absence of an EHR-based system. It would be valuable to study the distribution of pathogens and perhaps even resistance patterns, monitoring incidences over time, and assessing the impact of national infection prevention policies [27,28].

Our findings make a direct contribution to the exploration of opportunities and requirements for large-scale automated surveillance, including BSI surveillance on a European level guided by the European Centre for Disease Prevention and Control [29]. They are also highly relevant in the broader context of ongoing efforts to enhance the reusability of routine care data for secondary purposes, such as large-scale automated surveillance, with the European Health Data Space (EHDS), established under Regulation (EU) 2025/327, playing a substantial role in [30]. Under this framework, federated networks for automated surveillance can be developed, in which centrally designed analytical tools are applied locally to more detailed routine care data without leaving the hospital; only aggregated results are shared. As such, in addition to the identified challenges in data collection, issues related to data ownership, privacy and scalability could also be addressed. However, building a federated infrastructure requires, among other things, a robust architecture and effective governance [31].

Strengths of this study include the systematically evaluation of this potential surveillance system for HOB, following the full CDC evaluation guidelines. We included both quantitative methods, to evaluate the technical feasibility and validate surveillance results using detailed EHR and laboratory data as the reference, and qualitative research methods to include the opinions of a range of stakeholders.

A limitation of the study methodology was that outcome validation of HOB surveillance results was performed at hospital level due to privacy limitations, which precluded calculation of sensitivity and positive predictive value for individual patients, part of the evaluation guideline. Additionally, we have not validated the denominator data, i.e. number of hospital admissions, from the open-source dataset. However, since we did not observe large differences between 2021 and 2023, we expect the results to be reliable. As HOB surveillance is currently not standard practice in the Netherlands, hospital-specific reference data were limited to four hospitals where these data were collected in a research context. This leads to the possibility that other inconsistencies in laboratory policies could be present that were not identified in this study. An inventory of laboratory policies prior to implementation could reveal such inconsistencies.

## Conclusion

Our study suggests that this automated HOB surveillance based on the secondary use of readily available data from the Dutch laboratory-based AMR surveillance system could serve as a practical starting point for national automated surveillance of pathogen-related HOBs with little added effort from hospitals. However, the recording of hospital admission dates should be improved to identify HOBs reliably. Increases can be detected, serving as a signal for hospitals to conduct in-depth investigations. However, the IPC potential of HOB surveillance should be explored further. For automated HOB surveillance in general, attention should be paid to the correct interpretation of the source data and to the comparability of surveillance outcomes, as demonstrated by the impact of hospital and laboratory policies on the results.

## Data Availability

The data that support the findings of this study are available from the corresponding author, [TvdK], upon reasonable request.

## Supplementary Data

1035000 Supplement
