# Supplementary material for: National automated surveillance of hospital onset bacteraemia and fungaemia using data from the national antimicrobial resistance surveillance system: a retrospective exploratory evaluation, the Netherlands, 2018 to 2023
Source: Euro Surveill. 2026 Jul 16;31(28):2500881. doi: 10.2807/1560-7917.ES.2026.31.28.2500881 (PMC13379685; doi:10.2807/1560-7917.ES.2026.31.28.2500881)

Supplementary files

This supplementary material is hosted by *Eurosurveillance* as supporting information alongside the article" *National automated surveillance of hospital onset bacteraemia and fungaemia using data from the national antimicrobial resistance surveillance system: a retrospective exploratory evaluation, the Netherlands, 2018 to 2023",* on behalf of the authors, who remain responsible for the accuracy and appropriateness of the content. The same standards for ethics, copyright, attributions and permissions as for the article apply. Supplements are not edited by *Eurosurveillance* and the journal is not responsible for the maintenance of any links or email addresses provided therein.

Supplement S1. Interviewees

**Function**	**Number of interviewees**		
	National level	Local level	
		Large hospital	Smaller hospital
**Medical microbiologist**	1	1	1
**Epidemiologist**	1	1	
**Infection control practitioner**		3	1
**Internal medicine physician (specialization infectious diseases or intensive care)**		2	1

Supplement S2. Topic guide interviews

Introduction

1. *Interviewee*
    a. *Function, experience with (healthcare associated) infection surveillance, working experience*
2. *Interviewers*
    a. *Function, involvement in study*

[Show slide to introduce HOB, with HOB definition]

HOB as surveillance target

3. *Is HOB useful as surveillance target?*
    a. *Are (part of the) HOBs preventable? Which are preventable? Which are not?*

b. *Does a HOB surveillance provide data for action? Which actions?*
c. *Is the HOB definition from PRAISE suitable?*
    i. *Hospital-onset period of 2 days*
    ii. *Episode duration of 14 days*
    iii. *Criteria for common commensals*

4. *To what extent is it useful for national surveillance?*
    a. *What is the additional value?*
    b. *Does it provide data for action?*
    c. *Who are stakeholders?*
    d. *What are prerequisites?*
        i. *Timeliness*
        ii. *Source determination*
        iii. *Information about resistance of microorganisms*
    e. *How to increase usefulness?*
5. *To what extent is it useful for hospital-level surveillance?*
    a. *What is the additional value?*
    b. *Does it provide data for action?*
    c. *Who are stakeholders?*
    d. *What are prerequisites?*
    e. *How to increase usefulness?*

[Slides to explain the ISIS-AR data, results data technical evaluation (Table 2) and outcome validation (Figure 2), show national trends, stratified by hospital type (Figure 3A), and for the ICU specific (Supplement S4), and microorganism distribution (Figure 3B)]

ISIS-AR as data source for HOB surveillance

6. *Do you think that the ISIS-AR data are suitable for a national HOB surveillance?*
    a. *Timeliness: are the results timely enough?*
    b. *Level of detail: Which level of detail is preferred to present the results?*
        i. *Both on national and local level*
    c. *What do you think about the availability of the data?*
    d. *What do you think about the completeness/representativeness of the data?*
    e. *Is any additional information needed to include in this system?*

Closure

Supplement S3. Complete overview data quality of ISIS-AR data for HOB surveillance based on the PRAISE MDS

PRAISE MDS					ISIS-AR database	
Variable	Data type	Values or format (if applicable)	Optional?	Comments	Availability	Completeness
PATIENTS						
patientId	string		MANDATORY	Should be pseudonymised	Yes	100%
patientSex	string	M (male); F (female); X (not male not female); U (unknown)	MANDATORY		Yes	100%
patientBirthDate	date	YYYY/MM/DD	MANDATORY	For sharing purpose consider dummy	Partly	100% for birthyear and -month
patientDeathDate	date	YYYY/MM/DD	OPTIONAL		No	
BLOODCULTURES						
bcId	string		MANDATORY	A unique identifier of BC microorganism. Example compound sampleId+isolateNumber. Must add isolateNumber for unique identification purposes	Yes	100%
sampleId	string		MANDATORY	one sample can have multiple isolates	Yes	100%
patientId	string		MANDATORY	reference to Patient.patientId	Yes	100%
sampleDate	date		MANDATORY		Yes	100%
sampleWardId	string	YYYY/MM/DD	MANDATORY	Ward where culture was taken -> if not present in hospital data then ward where pt was admitted on day of culture; if pt was transferred on day of blood culture, take first ward (where patient came from)	Yes	100% for sample ward or order ward
isolateNumber	integer		MANDATORY		Yes	100%

bcMicroorgLocalId	string		MANDATORY	Local ID of the microorganism in lab system = basis for algo. Should curate this for example if determination for some species is unreliable (e.g. E. cloacae complex, B. cereus complex).	Yes	100%
attributableWardId	string		MANDATORY	Ward where pt was 2 days prior to culture taken if patient was transferred on day of blood culture, take first ward (where patient came from)	No	
admissionHospDate	date		MANDATORY		Partly	64 % of the isolates have a admission date<br><br>79 % of all ICU isolates have an admission date
DENOMINATOR	(aggregated data)	YYYY/MM/DD		Denominator (calculated by data provider)<br>These are denominator data of all patients wardGroup under analysis. See examples below		
wardGroupType	string		MANDATORY	Defines at what level the analysis will be done, for example enter hospital, individual wards, local ward groups or ECDCWard groups	Yes	Hospital-wide and ICU
wardGroupValue	string	value_set<br><br>HOSPITAL<br><br>WARD<br><br>LOCALWARD<br><br>ECDCWARD	MANDATORY	Specifies the value options for the denominators.	Yes	
patientDays	numeric	value_set containing a values applicable to wardGroups	MANDATORY	Number of patient days in wardGroupValue within specified period type.<br>Midnight method: assign patient day to ward where patient was at 00:00	Partly	2018 – 2021
periodType	value_set		MANDATORY	If possible, DAY, else the lowest level feasible.	Yes	Yearly

calendarDateStart	date	Valueset<br><br>DAY<br><br>MONTH<br><br>QUARTER<br><br>YEAR	MANDATORY	Start date to which the denominator value applies. If Period type is DAY, then calendarDateStart = calendarDateEnd	Yes	
calendarDateEnd	Date	YYYY/MM/DD	MANDATORY	end date to which the denominator value applies (inclusive). If Period type is DAY, then calendarDateStart = calendarDateEnd	Yes	
numberOfAdmissions	numeric	YYYY/MM/DD	MANDATORY	Number of admissions to that specific WardGroupValue on specified period type	Partly	Available in period 2018-2021
numberOfBloodCultureSamples	numeric		MANDATORY	Count distinct sampleID If available, else try to submit all BC's according to stage 1 (sampleWard, also for negative cultures)	No	No negative cultures available

Supplement S4. Stability and representativeness of the Dutch Infectious Disease Surveillance Information System for Antimicrobial Resistance data and data suitable for hospital-onset bacteraemia and fungaemia surveillance, the Netherlands, 2018–2023

		ISIS-AR data 2018-2023		Data eligible for HOB surveillance 2018-2023 (>80% of admission dates available)	
**CDC guideline concept**	**Characteristic**	**Total ISIS-AR data**	**Selection of hospitals consistently included in ISIS-AR data2**	**Annual surveillance-suitable data**	**Selection of hospitals with consistent surveillance-eligible data***
	**Number of records (n)**	428,360	388,114	171,454	136,175
**Stability**	**Number of hospitals**		54 (8 academic, 26 top-clinical and 20 general hospitals)		14 (6 academic, 4 top-clinical and 4 general hospitals)
	2018	64		32	
	2019	60		26	
	2020	66		33	
	2021	65		32	
	2022	66		30	
	2023	71		28	
**Representativeness (blood culture isolate data)[1]**	**Birth year** (median (q1-q3))	1950 (1941-1963)	1950 (1941-1963)	1954 (1944-1969)	1955 (1945-1973)
	**Sex** (% (n))				
	Males	60 (256,273)	60 (232,371)	61 (104,569)	61 (82,427)
	Females	40 (172,085)	40 (155,741)	39 (66,885)	39 (53,748)
	Other	0 (2)	0 (2)	(0)	0 (0)
	**Hospital type** (% (n))				
	General	26 (110,481)	25 (98,560)	17 (28,445)	13 (17,748)
	Top-clinical	49 (210,440)	50 (195,530)	43 (73,921)	35 (47,141)
	Academic	25 (107,344)	24 (94,024)	40 (69,012)	52 (71,286)
	Unknown	0 (95)	0 (0)	0 (76)	0 (0)
	**Ward type** (% (n))				
	ICU	15 (64,739)	15 (57,530)	18 (30,494)	20 (26,752)
	Non-ICU	85 (363,596)	85 (330,569)	82 (140,957)	80 (109,423)
	Not applicable	0 (25)	0 (15)	0 (3)	0 (0)
	**Medical specialty** (% (n))				
	Internal medicine	28 (121,189)	27 (105,586)	23 (40,005)	20 (26,572)
	ICU	9 (38,460)	9 (35,195)	10 (17,842)	12 (16,345)
	Surgery	9 (36,882)	9 (33,954)	8 (14,362)	9 (12,758)
	Pulmonary diseases	7 (29,789)	7 (27,120)	6 (9,984)	5 (7,053)
	Emergency department	7 (29,524)	7 (25,918)	8 (13,157)	4 (5,453)
	Cardiology	5 (22,210)	5 (19,659)	5 (8,694)	4 (5,666)
	Urology	4 (18,683)	4 (17,209)	3 (5,755)	3 (4,160)

	Gastro-enterology	4 (17,777)	4 (16,391)	4 (6,931)	4 (5,396)
	Oncology	3 (11,718)	3 (11,718)	5 (9,389)	6 (8,639)
	Paediatrics	3 (11,600)	3 (10,578)	3 (4,440)	3 (4,759)
	Neurology	3 (10,716)	2 (9,576)	2 (3,658)	2 (2,645)
	Haematology	2 (9,132)	2 (9,129)	3 (5,926)	6 (7,877)
	Other	17 (70,680)	17 (66,081)	18 (31,311)	21 (28,852)

CDC: Centers for Disease Control and Prevention; HOB: hospital-onset bacteraemia and fungaemia; ICU: intensive care unit; ISIS-AR: Dutch Infectious Disease Surveillance Information System for Antimicrobial Resistance; Q: quartile.

[1]Percentages based on all positive blood culture isolates from the study period

[2]Consistent data: data continuously available in all 6 years of study period

All differences between the total ISIS-AR dataset and the annual surveillance suitable data, and between the selection of hospitals consistently included in ISIS-AR data and selection of hospitals with consistent surveillance-eligible data are statistically significant (p<0.0001).

<u>Supplement S5</u>. Differences per micro-organism group per hospital for HOBs calculated based on ISIS-AR data and data directly from the electronic health record (reference data)

Differences ranged between -18.8 – 5.8% for Enterobacterales (four year average per hospital, HOB-EHR reference); -7.2 – 1.6% for Enterococci; and 4.3 – 29.8% for *Staphylococcus aureus*. Larger discrepancies were observed in the number of HOBs caused by CNS in hospital 1 and 2. Differences for CNS ranged between -87.3 – -2.3% and between -51.7 – 6.8 for Streptococci.

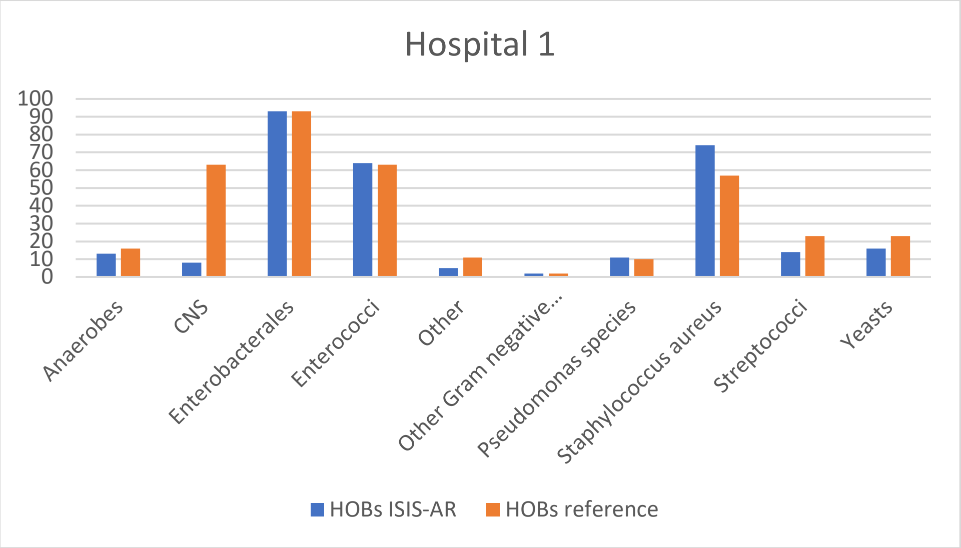

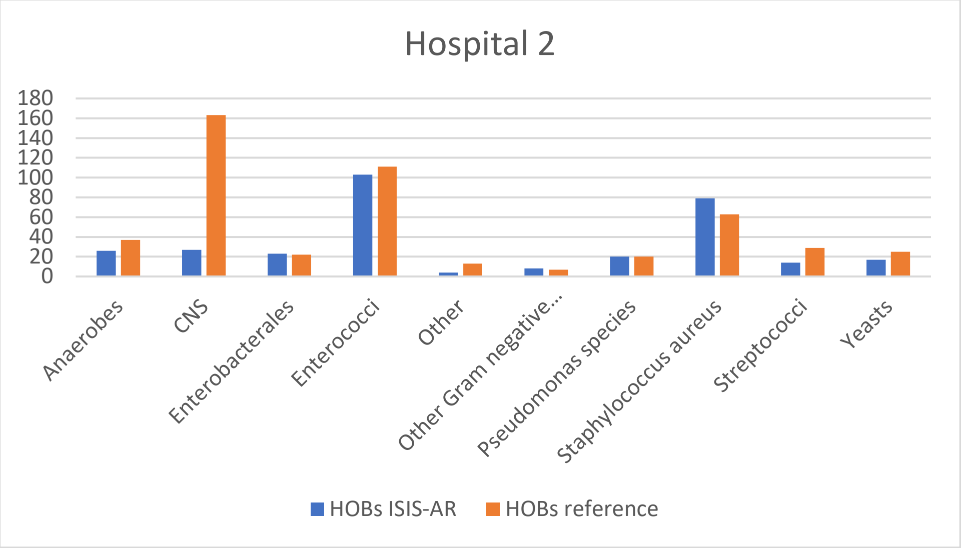

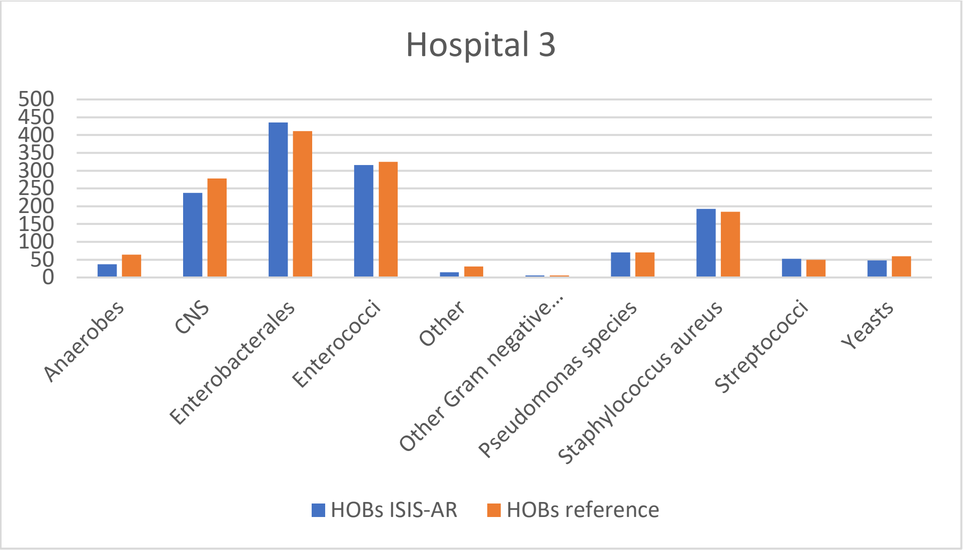

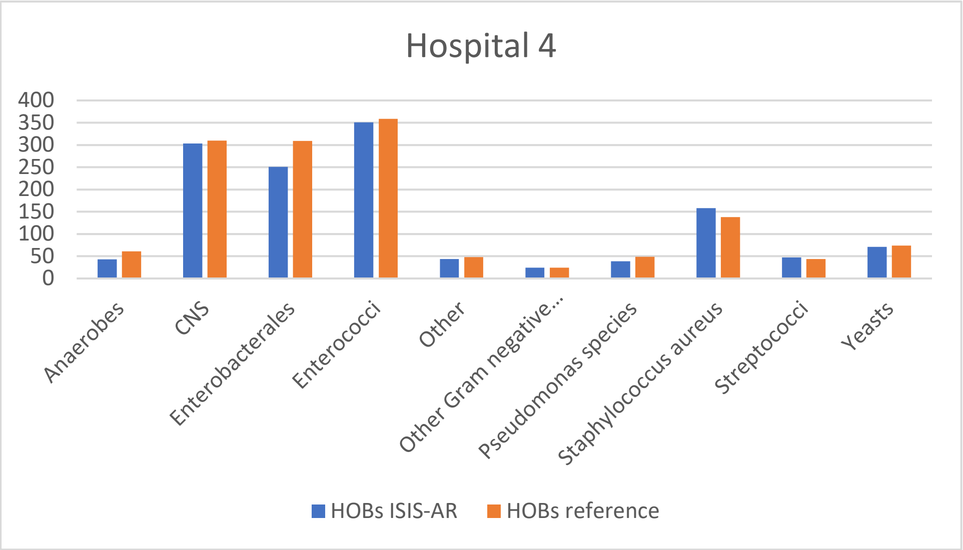

Supplement S6. ICU-specific HOB trends caused by pathogens, stratified by hospital type

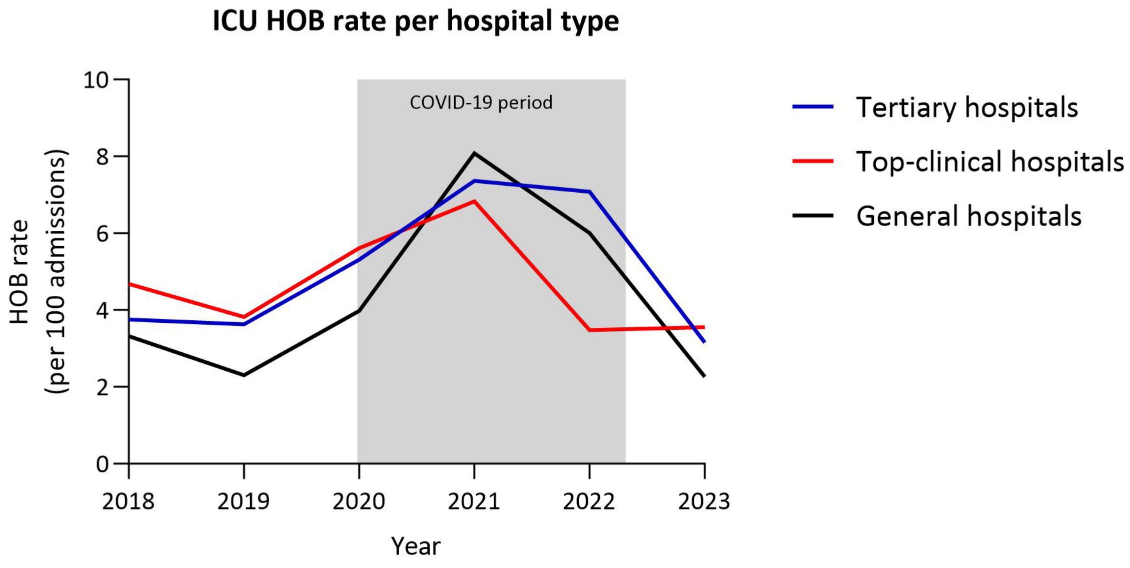

Supplement: Supplement [file 25-00881_vdKOOI_Supplement.pdf]
